# Supplementary material for: Profile of the Nicotinic Cholinergic Receptor Alpha 7 Subunit Gene Expression is Associated with Response to Varenicline Treatment
Source: Genes (Basel). 2020 Jul 6;11(7):746. doi: 10.3390/genes11070746 (PMC7397196; doi:10.3390/genes11070746)
Supplement: Supplementary file 1 [file genes-11-00746-s001.pdf]

Profile of the nicotinic cholinergic receptor alpha 7 subunit gene expression is associated with response to varenicline treatment

Juliana Rocha Santos[1], Paulo Roberto Xavier Tomaz[1], Jaqueline Scholz[2], Patrícia Viviane Gaya[2], Tânia Ogawa Abe[2], José Eduardo Krieger[1], Alexandre Costa Pereira[1], Paulo Caleb Júnior Lima Santos[3*]

[1] Laboratory of Genetics and Molecular Cardiology, Instituto do Coracao (InCor), Hospital das Clinicas HCFMUSP, Faculdade de Medicina, Universidade de Sao Paulo, Sao Paulo, Brazil.
[2] Smoking Cessation Program Department, Instituto do Coracao (InCor), Hospital das Clinicas HCFMUSP, Faculdade de Medicina, Universidade de Sao Paulo, Sao Paulo, Brazil.
[3] Department of Pharmacology – Escola Paulista de Medicina, Universidade Federal de Sao Paulo, EPM-Unifesp, Sao Paulo, Brazil.

Supplementary table 1 - Median values of ΔCT genes according with time periods and outcome groups

	Resistant T0		Resistant T2		Resistant T4	
	**ΔCT**	**IC 95%**	**ΔCT**	**IC 95%**	**ΔCT**	**IC 95%**
CHRNA5	8.18	(7.32 – 8.70)	8.45	(7.48 – 8.87)	7.67	(7.27 – 9.05)
CHRNA7	6.62	(6.17 – 6.97)	8.02	(7.07 – 8.48)	7.19	(6.96 – 7.86)
CHRNG	6.20	(5.79 – 6.95)	6.47	(6.01 – 6.68)	6.63	(6.02 – 7.02)
COMT	4.67	(4.40– 5.01)	4.80	(4.43 – 5.03)	4.87	(4.52 – 5.24)

	Success T0		Success T2		Success T4	
	**ΔCT**	**IC 95%**	**ΔCT**	**IC 95%**	**ΔCT**	**IC 95%**
CHRNA5	8.32	(7.38 – 9.17)	7.07	(6.53 – 8.96)	8.37	(7.54 – 8.82)
CHRNA7	7.26	(6.11 – 8.42)	7.04	(6.40 – 7.79)	7.38	(6.76 – 8.20)
CHRNG	6.82	(6.19 – 7.74)	6.83	(6.56 – 7.33)	6.59	(6.25 – 7.03)
COMT	4.88	(4.30 – 5.11)	4.58	(4.33 -5.15)	4.81	(4.48 – 5.16)

$\Delta CT = (CT_{\text{target gene}} - CT_{\text{housekeepings genes mean}})$. T0 = initial time before pharmacological treatment, T2 = two weeks after pharmacological treatment, T4 = four weeks after pharmacological treatment. 95% CI = 95% confidence interval.

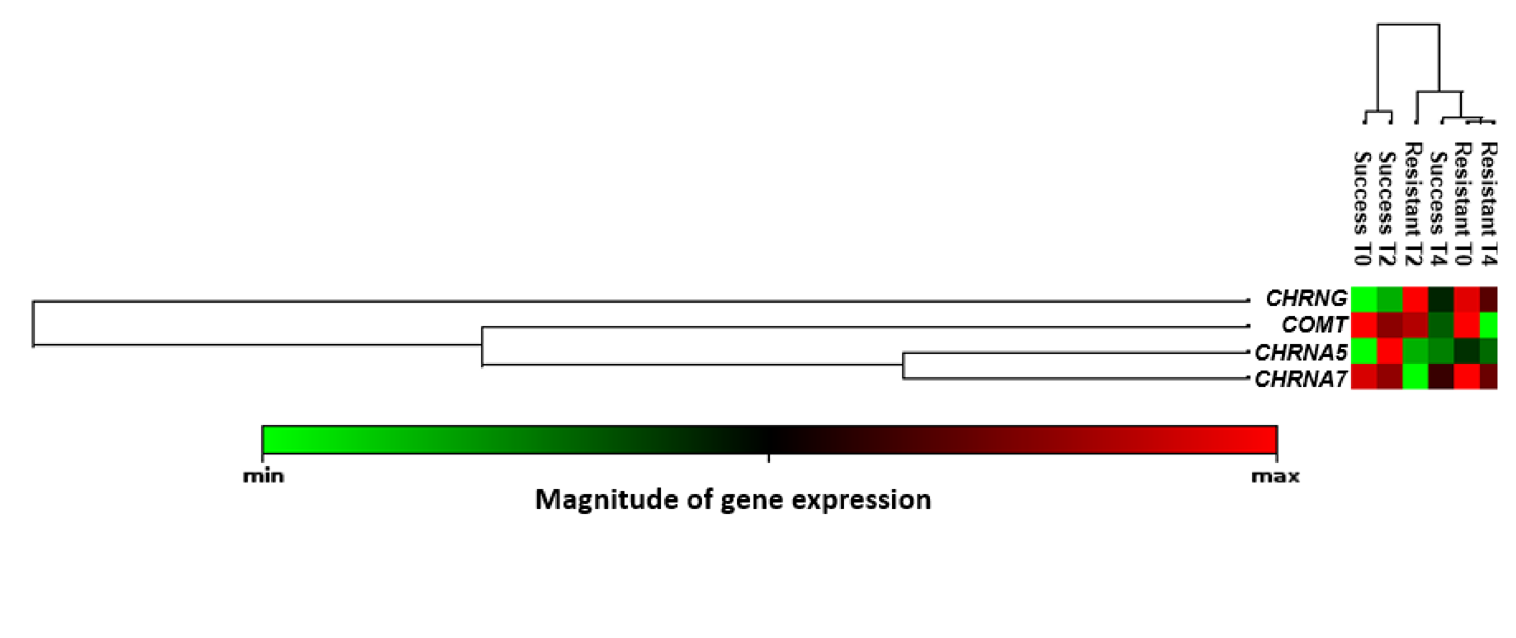

Supplementary figure 1 - Heat map of gene expression. The figure shows the magnitude of gene expression between the groups. The lighter color (green) indicates less expression and the darker color (red) indicates greater expression. T0 = initial time before pharmacological treatment, T2 = two weeks after initiation of pharmacological treatment, T4 = four weeks after initiation of pharmacological treatment.

Supplementary panel 1 - *Housekeeping genes.*

Genes	Encoded protein
B2M	Beta-2-microglobulin
GAPDH	Glyceraldehyde-3-phosphate dehydrogenase
HPRT1	Hypoxanthine phosphoribosyltransferase 1
ACTB	Actin beta

Supplementary panel 2 - Genes chosen for expression assay

Genes	Encoded protein
CHRNA3	Neuronal acetylcholine receptor subunit alpha-3
CHRNA4	Neuronal acetylcholine receptor subunit alpha-4
CHRNA5	Neuronal acetylcholine receptor subunit alpha-5
CHRNA6	Neuronal acetylcholine receptor subunit alpha-6
CHRNA7	Neuronal acetylcholine receptor subunit alpha-7
CHRNB2	Neuronal acetylcholine receptor subunit beta-2
CHRNB3	Neuronal acetylcholine receptor subunit beta-3
CHRNB4	Neuronal acetylcholine receptor subunit beta-4
CHRNG	Acetylcholine receptor subunit gamma
DRD1	Dopamine receptor D1
DRD2	Dopamine receptor D2
DRD3	Dopamine receptor D3
DRD4	Dopamine receptor D4
HTR3A	5-hydroxytryptamine receptor 3[a]
HTR3B	5-hydroxytryptamine receptor 3B
COMT	Catechol-O-methyltransferase
SLC6A3	Solute carrier family 6 member 3 (DAT protein)
